# Fragment assignment in the cloud with eXpress-D

**DOI:** 10.1186/1471-2105-14-358

**Published:** 2013-12-07

**Authors:** Adam Roberts, Harvey Feng, Lior Pachter

**Affiliations:** 1 Department of Computer Science, 387 Soda Hall, UC Berkeley, Berkeley, CA 94720, USA; 2 Departments of Mathematics and Molecular & Cell Biology, 970 Evans Hall, UC Berkeley, Berkeley, CA 94720, USA

## Abstract

**Background:**

Probabilistic assignment of ambiguously mapped fragments produced by high-throughput sequencing experiments has been demonstrated to greatly improve accuracy in the analysis of RNA-Seq and ChIP-Seq, and is an essential step in many other sequence census experiments. A maximum likelihood method using the expectation-maximization (EM) algorithm for optimization is commonly used to solve this problem. However, batch EM-based approaches do not scale well with the size of sequencing datasets, which have been increasing dramatically over the past few years. Thus, current approaches to fragment assignment rely on heuristics or approximations for tractability.

**Results:**

We present an implementation of a distributed EM solution to the fragment assignment problem using Spark, a data analytics framework that can scale by leveraging compute clusters within datacenters–“the cloud”. We demonstrate that our implementation easily scales to billions of sequenced fragments, while providing the exact maximum likelihood assignment of ambiguous fragments. The accuracy of the method is shown to be an improvement over the most widely used tools available and can be run in a constant amount of time when cluster resources are scaled linearly with the amount of input data.

**Conclusions:**

The cloud offers one solution for the difficulties faced in the analysis of massive high-thoughput sequencing data, which continue to grow rapidly. Researchers in bioinformatics must follow developments in distributed systems–such as new frameworks like Spark–for ways to port existing methods to the cloud and help them scale to the datasets of the future. Our software, eXpress-D, is freely available at: http://github.com/adarob/express-d.

## Background

Modern sequencing experiments usually involve the shearing of DNA or cDNA into relatively short fragments for processing on a high-throughput sequencing device, such as the Illumina HiSeq. In the analysis of the resulting data, one of the first steps is to align the reads representing these partially-sequenced fragments to a set of target sequences. This procedure identifies locations within the target sequences from which each fragment may have originated using a threshold on mismatches and insertions or deletions (indels), thus reducing the focus of downstream analysis to only highly probable loci. Numerous read mappers exist to solve this problem with various features and performance characteristics, the most popular of which are based on the Burrows-Wheeler transform [1,2].

A common problem in downstream analysis of the resultant alignment data is that fragments often map ambiguously to multiple target sequences. For example, in the case of RNA-Seq, a given fragment might align to multiple isoforms of a gene as well as to multiple genes within a gene family. This ambiguity makes it difficult to measure the abundance of transcripts, especially those with few unique regions. A similar problem occurs with ChIP-Seq data, where fragments align to many regions of the genome, complicating the problem of peak finding for determining binding sites [3]. Another example is in metagenomics, where researchers wish to detect the presence and relative abundance of various closely related species of microorganisms in a pooled sample of DNA [4].

### Previous approaches

The earliest solution for this problem was to ignore any fragments that align ambiguously. However, such techniques discard large amounts of useful information and can lead to significant biases in the analysis. For example, we observe over 25% of fragments being ambiguous in recent RNA-Seq experiments in the human transcriptome.

Mortazavi *et al.*[5] provided a better solution the problem, later named the rescue method by [6], through which ambiguous fragments are assigned proportionally to their potential origins based on the initial gene abundances computed from unique fragment counts. This idea, based on the oft-valid assumption that genes that generate more unique fragments are likely to also generate more ambiguous fragments, was extended by both Li *et al.*[6] and Trapnell *et al.*[7] after noting that rescue was equivalent to a single iteration of the *expectation-maximization* (EM) algorithm for a simple model of RNA-Seq. In the full version of the algorithm, reads are probabilistically assigned in an *expectation* (E) *step* based on the current abundance (and possibly other experimentally related) parameter estimates. These estimates are then updated in a *maximization* (M) *step* to those that maximize the likelihood given the assignments, which in this case is proportional to the number of assigned fragments per target. These steps are repeatedly alternated and are guaranteed to improve the likelihood at each iteration. Since the likelihood function in this simple model is concave, the estimates will eventually converge to the maximum likelihood solution.

Several probabilistic models have since been introduced, building off still earlier models [8] to include parameters for features such as fragment length, indel and substitution errors, and sequence-specific biases [9]. Furthermore, various methods have been proposed for optimizing the likelihood of a given model. The EM solution has been the most successful to date, but has failed to scale with the rapid growth in the size of typical sequencing datasets [10]. The large number of iterations required for convergence of the EM algorithm means that details of the alignments must be stored in memory for fast access, since reading them from disk thousands of times would be intractable. In response to the large memory requirements, heuristics and approximations have been introduced to reduce the footprint of these methods.

**Figure 1:**
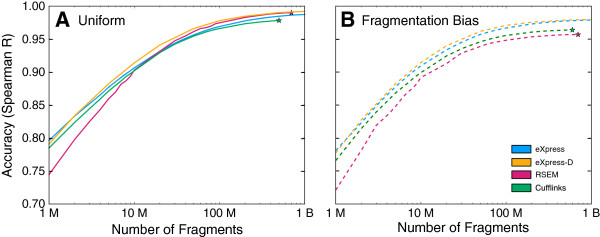
**Accuracy Comparison for Synthetic Data.** Accuracy of eXpress, eXpress-D, RSEM, and Cufflinks at multiple sequencing depths in a simulation of a billion fragments (read pairs) generated with **(B)** and without **(A)** sequence-specific bias. Expanded from Figure 2 in [10] and produced using the same synthetic datasets. Each algorithm was presented the same multisized subsets of 1 billion simulated fragments and the Spearman ranked correlation coefficient was calculated between the resulting estimates and ground-truth abundance values used in the simulation. The stars represent where each of the software packages crashed or were halted due to the test machine’s memory constraint (512 GB for all but eXpress-D).

The developers of RSEM[11] have been conservative in their attempts to scale. By ignoring fragments that align ambiguously to a large number of transcriptomic locations (200 by default), the memory requirements are somewhat reduced, along with the number of iterations. Using what is essentially the full batch EM algorithm allows RSEM to retain high accuracy when ignoring deficiencies in bias modeling ([10] and Figure 1A) but makes scaling a challenge.

eXpress[10] follows a model very similar to RSEM, but side-steps the scaling issue by using an alternative optimization procedure–the online EM algorithm–that only requires a small, constant amount of memory. The online–or streaming–EM algorithm approximates the batch algorithm used in RSEM without the need to consider any of the alignments more than once. Therefore, the alignments of each fragment can be read from disk once, processed, and then discarded from memory. This leads to huge reductions in the memory and time requirements of the method, but causes it to be somewhat less accurate than RSEM when eXpress’s bias modeling is disabled ([10] and Figure 1A).

Another approach to reducing the memory footprint is to exploit the structure of the likelihood function. [12] introduces the *ambiguity graph* for a set of transcripts, or other target sequences. In this graph, each target is represented as a node and edges are added between pairs of nodes when an ambiguous fragment aligns to both. Targets in different components of this graph are independent of each other, providing a decomposition of the likelihood function. Thus, the fragments and targets making up separate components can be optimized serially, reducing the memory requirements to the size of the largest component. While this component often makes up a significant fraction of the data, graph partitioning techniques can be used to further reduce the block sizes with little effect on accuracy. However, this method has not been implemented as part of a batch EM solution to date.

Cufflinks[7] approximates the above procedure by partitioning the fragments and targets based on their genomic positions. Since ambiguous alignments in RNA-Seq are commonly due to equivalent mappings to multiple isoforms, Cufflinks determines the maximum likelihood assignment of fragments for each non-overlapping genomic locus using the EM algorithm. Fragments that are not unique to a single locus are initially assigned uniformly to the ambiguous loci, but can be better assigned in a second iteration of Cufflinks using a method similar to rescue. This method essentially assumes that the components of the ambiguity graph strictly correspond to genomic loci, which is, in fact, rarely the case [5]. In [10], we provide evidence that this assumption is the source of Cufflinks lower accuracy on simulated data when compared to RSEM and eXpress.

### To the cloud

While these solutions have all used algorithms and heuristics to deal with bounded computer memory resources, another approach is to handle the increasing size of datasets by scaling up the compute resources. It is currently infeasible for every small lab to purchase machines with enough RAM to fully analyze today’s datasets using the batch EM algorithm. However, large clusters of compute nodes are now available for relatively low cost with pay-by-use cloud platform services, such as Amazon’s Elastic Compute Cloud (EC2) [13]. Developing software to take advantage of the distributed resources on clusters of commodity machines is nontrivial, as issues such as failure recovery and communication must be dealt with [14].

MapReduce is an abstraction that allows developers to access the power of large distributed commodity clusters without having to explicitly handle details such as data partitioning, work scheduling, and software and hardware failures. The MapReduce programming model involves a series of calls to primitive map and reduce methods, with reordering and grouping allowed between. MapReduce was originally conceived by Google [15] in conjunction with the Google File System (GFS) [16], a fault tolerant, distributed file system–the “disk” that MapReduce utilizes. Both inspired open-source counterparts that compose the core Apache Hadoop project: Hadoop MapReduce and the Hadoop Distributed File System (HDFS) [17].

Myrna[18] applies Hadoop MapReduce to the analysis of RNA-Seq data, using Hadoop to count the unique alignments in an experiment. The map phase iterates through the alignments, emitting a tuple identifying the transcript that each fragment is aligned to. In the reduce phase, the unique alignments for each transcript are accumulated to produce the total counts. Since the fragments can be processed independently in the map phase, Hadoop can distribute the fragments randomly to multiple nodes. In the reduce phase, Hadoop can be set to automatically assign tuples for each transcript to the same node, allowing the accumulations to occur in an independent, distributed manner.

This method could be also extended to handle ambiguous mappings by implementing the EM algorithm using many iterations of MapReduce. The map phase would correspond to the E-step, in which a tuple is emitted for each alignment specifying the target and the probability that it is origin of the fragment based on the likelihood model and a set of global parameter estimates. The reduce phase would correspond to the M-step, in which the probabilistic assignments would be accumulated and the values normalized to produce the updated maximum likelihood parameters estimates for use in the subsequent iteration.

However, the problem with implementing the EM algorithm using Hadoop MapReduce is that the system is not tailored for data reuse. In Hadoop, the dataset being scanned is re-read from disk before every map step, and the results of intermediate computations are written to temporary files after the map. In our application, EM would be implemented as a map task. This means that the alignments would have to be loaded from disk before the E-step and a partial set of probabilistic assignments written to disk after the M-step. Then, on a single node executing a reduce task, partial sets of probabilistic assignments would be fetched from the temporary files on map nodes and loaded into memory for rendezvous and normalization. An on-disk file containing likelihood parameters would also be updated during this reduce step. Thus, numerous disk operations done in Hadoop’s map and reduce tasks would create a significant bottleneck.

The approach we take instead is to use Apache Spark, an open-source framework that provides in-memory, fault-tolerant cluster computing by implementing *resilient distributed datasets* (RDDs) [19]. Spark is an alternative compute engine to Hadoop that implements the MapReduce abstraction by allowing users to apply map and reduce functions over RDDs. In conjunction with the a distributed file system–such as GFS, HDFS, or Amazon’s S3–Spark handles all issues of fault tolerance and partitioning across the cluster nodes. Unlike MapReduce, however, once a subset of the data is read from the filesystem into memory, it can be made to persist in the RAM of the compute nodes, allowing an application to efficiently scan it throughout many iterations.

Furthermore, Spark provides two types of shared variables based on common use cases that are well-suited for the workflow of the EM implementation: broadcast variables and accumulable variables. A broadcast variable is a read-only piece of data that is distributed to all worker nodes. An accumulable variable references an append-only data structure that is updated by each worker node’s local process and then fully combined by the process running on the master node. Broadcasted and append-only data structures both persist in-memory. eXpress-D utilizes these shared variables to distribute and update parameters and accumulate probabilistic assignments. The following section contains more detail on the implementation.

When given enough RAM, consecutive map executions, broadcasts, and accumulations can avoid disk spilling, which makes Spark particularly appropriate for the EM algorithm [20]. By implementing the EM algorithm for ambiguous fragment assignment using Spark, in conjunction with Amazon S3 for persistent storage, we can easily scale the method to very large datasets by combining the resources of multiple compute nodes, providing in-memory storage of alignment data while also taking advantage of large-scale parallel computations.

## Method

### Model

Our implementation maximizes the likelihood of the generative model presented in [10].

### Preprocessing with eXpress

By default, the distributed file system partitions a dataset stored as text using line breaks to delineate discrete units of processing. In our case, a discrete unit is the collection of alignments of a single fragment for the alignment file, and the name and sequence of a single target for the target file. Since the commonly used formats for alignments and targets (SAM and FASTA, respectively) do not conform to this standard, we must pre-process the files to produce inputs that can be partitioned by the file system. At the same time, we wish to make our input files as small as possible to reduce the time required for network transfers.

To achieve these goals, we modified eXpress–which has already been optimized for parsing the standard SAM and FASTA files–to produce input files compatible with our method. The format of these new files are newline-delimited, serialized Protocol Buffers, which are encoded in base64 to ensure no newline characters appear in the serialization itself. The Protocol Buffer specification is shown in Table 1 for both alignments and targets. We have avoided including any unnecessary or redundant information and compressed nucleotide sequences to byte arrays, requiring approximately 2 bits per nucleotide. The resulting files are significantly smaller than the original binary SAM (BAM) and FASTA files.

**Table 1:** Protocol Buffer specifications

**Field**	**Type**	**Description**
Fragment		
name	string	Unique query name of fragment in SAM file
paired	bool	Boolean specifying if both ends were sequenced
alignments	FragmentAlignments	Collection of alignments for fragment
FragmentAlignment		
target_id	uint32	ID of target aligned to (index in SAM header)
read_l	ReadAlignment	Alignment information for 5’ (left) read, if exists
read_r	ReadAlignment	Alignment information for 3’ (right) read, if exists
ReadAlignment		
first	bool	Boolean specifying if this end was sequenced first
left_pos	unit32	0-based left endpoint of alignment to reference
right_pos	unit32	0-based right endpoint of alignment to reference
mismatch_indices	byteArray	Positions in read that differ from reference
mismatch_nucs	byteArray	Nucleotides in read at mismatches, 2 bits/nuc
Target		
name	string	Unique name of target sequence
id	uint32	Index of target in SAM header
length	uint32	Number of nucleotides in target sequence
seq	byteArray	Nucleotides of target sequence, 2 bits/nuc

eXpress pre-processes the input data (SAM/BAM and FASTA file) and converts it to a format that is compatible with the distributed file system’s partitioning scheme. The information for each target and fragment are put into a space-efficient Protocol Buffer–retaining only the information necessary for optimization–, which is then serialized and encoded in base64. Each target or fragment takes up exactly one line in the file created for input into eXpress-D.

**Figure 2:**
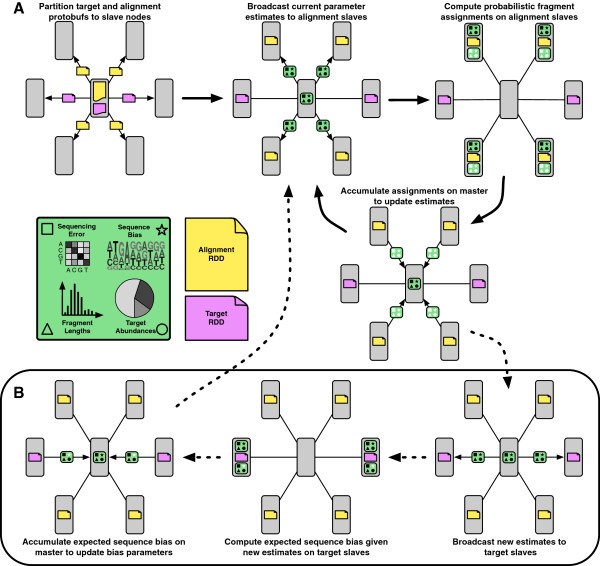
**Method overview.** The top portion **(A)** shows the procedure for running the distributed batch EM algorithm with Spark, ignoring sequence-specific bias. First, blocks of partitioned alignments (yellow) and targets (magenta) are distributed to the slave nodes, stored in memory as RDDs, and cached. An initial set of parameter estimates (green with black symbols) are broadcast to the slaves with alignments. The alignments on each slave are probabilistically assigned and new parameter estimates are partially accumulated (green with white symbols) on each node. These are then sent back to the master to be fully combined and re-broadcast for the next round. When sequence-specific bias is enabled, additional processing **(B)** takes place between some rounds. The parameter estimates are sent to the slaves with targets and the expected sequence bias given the current abundance estimates are accumulated and normalized on the master node to produce updated weights [9]. Auxiliary parameters (error, bias, and fragment lengths) are fixed after 1000 rounds, and the estimation procedure stops when convergence of the abundance parameters is reached.

Once the input files are loaded into HDFS or S3 on the cluster, our application can be run on Spark to begin fragment assignment. Figure 2 outlines the procedure, which is described in more detail in the following subsections.

### Preprocessing on Spark

The input files are parsed by Spark and loaded into the memory of the slave nodes as RDDs. The per-alignment indices for accessing the relevant elements of the error and bias Markov chain parameter matrices are then precomputed and stored in a transformed RDD. Each partition of the transformed RDD is approximately 700 megabytes and stores about 1 million fragments.

### Processing without bias correction

The algorithm for processing without bias correction is depicted at the top of Figure 2. The current target abundance, error substitution Markov chain, and fragment length distribution estimates (all initially set to be uniform) are broadcast to the slave nodes storing alignment RDDs. Given these distributions, the fragments on each slave are probabilistically assigned to the aligned targets using the likelihood function from [10]. The appropriate categories of the latent distributions are incremented by the posterior probabilities of the assignments at each slave node to produce new empirical distributions. These counts are then accumulated by the master node and Laplace smoothing is applied before they are normalized. The updated parameter estimates are then broadcast to the slave nodes and the procedure is repeated until convergence is detected (see below).

### Processing with bias correction

Previous work demonstrates that significant improvements in accuracy can be attained by modeling sequence-specific bias [9,11]. We have included a bias correction mode (enabled by default) to take advantage of these improvements, as illustrated at the bottom of Figure 2. The primary algorithm remains the same as outlined above with the addition of Markov chain parameters modeling the sub-sequences surrounding the 5’ and 3’ fragment ends. Estimates of these parameters are broadcast to the slaves, used in the likelihood calculation, and updated empirically, similar to the other hidden parameters. Instead of probabilities, the bias parameters are ratios of the observed to expected frequencies of these sub-sequences and are used as weights in the likelihood function. The observed frequencies are accumulated empirically along with the other parameters as described above, but the expected frequencies must be computed by sliding windows along the target sequences and counting the occurrences of various sub-sequences weighted by the current target abundance and fragment length parameter estimates. To make these repeated updates efficient, we broadcast the current model parameter estimates to the slave nodes storing target RDDs and have them compute local frequencies based on sliding windows over the RDDs in memory. The frequencies are then accumulated by the master node, allowing the bias weights to be updated before the next iteration.

### Freezing of auxiliary parameters

We define the auxiliary parameters to be all parameters of the model except for the target abundance parameters, which are the main parameters of interest. There are two reasons for freezing the auxiliary parameters after a suitable number of iterations: 

1. The auxiliary parameters can be estimated accurately much earlier than the target abundance parameters since they are fewer in number. Otherwise the algorithm will be wasting a significant amount of time unnecessarily updating their distributions at later iterations.

2. The model is only convex given fixed auxiliary parameters. Since we repeat the EM steps until convergence is reached, we want a guarantee that processing will not continue indefinitely. Given fixed auxiliary parameters, the likelihood function is log-linear, and the EM algorithm is guaranteed to converge to the maximum likelihood solution.

We therefore have chosen to use the following auxiliary parameter update scheme: The parameters are updated at every iteration for the first 20 and are then only updated every 100 iterations until 1000 iterations are reached, at which point they are frozen.

### Numerical stability

To avoid underflow, all probabilities distributions are logged before being used in likelihood computations, which has the extra benefit of allowing the use of faster additions instead of multiplications. The assignment probabilities are exponentiated before incrementing the empirical distributions, since there is no concern of numerical instability in the update step.

### Convergence detection

We halt the algorithm when convergence of the target sampling probabilities is detected in a manner similar to [6]. The parameters are considered to have converged when all targets with a sampling probability of at least 10^−7^ have a relative change of no more than 10^−2^ between two consecutive iterations.

## Results and discussion

### Test data

In order to test the performance of eXpress-D in terms of both speed in accuracy and in comparison with previous methods, we chose to use the two synthetic datasets from [10]. Both datasets contain a billion fragments that were simulated according to the generative model described in [10], one including sequence-specific bias and one without. Alignment was done with Bowtie v0.12.7 and TopHat v2.0.0 (using Map2GTF), allowing for three mismatches in both cases.

### Method comparisons

We compared performance of eXpress-D with RSEM, Cufflinks, and eXpress using the results generated in [10]. In that analysis RSEM v1.1.11, Cufflinks v1.4.0 (with -u), and eXpress v1.2.0 were used. We have provided scripts for repeating this analysis in conjunction with the data published with [10] Additional file 1.

### Cluster and experiment setup

For running experiments, we used Amazon EC2 clusters comprising m3.2xlarge instances, each of which has 8 virtual CPUs and 30 GB of memory [21]. A virtual CPU is rated at 3.25 EC2 Compute Units (ECU), which is roughly equivalent to a 1.0–1.2 GHz 2007 Xeon processor [22]. Even though 30 GB may seem excessive, we found that it was necessary to avoid full, costly garbage collection runs by the Java Virtual Machine (JVM) that Scala runs on, which could delay each iteration by tens of seconds.

A cluster was launched for subsets of various sizes of each test dataset. Starting from 3 slave nodes used for 50 million and fewer fragments, the number of slave nodes used increases proportionally with the dataset size, until we reach 60 slave nodes used for 1 billion fragments. Each set of fragments is broken down to partitions of approximately 1 million fragments, the size of which is 128 MB when stored on disk and 700 MB when stored in memory as Java objects. The partitions are stored using Amazon’s S3 persistent store, and for eXpress-D executions is cached on a slave assigned by the Spark scheduler. To measure how runtimes scale with increasing dataset sizes and cluster resources, we executed eXpress-D four times on each cluster for every dataset and report the average of those runs on that cluster. Furthermore, runs over datasets simulated with and without bias were done sequentially on the same cluster. We also used only trials where no Spark processes were interrupted due to disconnected instances, or other machine component failures.

### Performance comparisons

Figure 1 reveals that eXpress-D outperforms all other methods compared for data simulated both with and without bias. This is unsurprising since it combines the exact generative model with the full batch EM algorithm for optimization, while the other methods make various approximations in one or the other. eXpress and Cufflinks use a complete model including bias, but eXpress optimizes with the *online* EM algorithm and Cufflinks assumes independence between genomic loci. RSEM optimizes with the batch EM algorithm but does not model sequence-specific bias. These approximations are made to help the algorithms process large datasets on a single machine, but by taking advantage of the cloud, eXpress-D does not need to sacrifice accuracy to scale.

**Figure 3:**
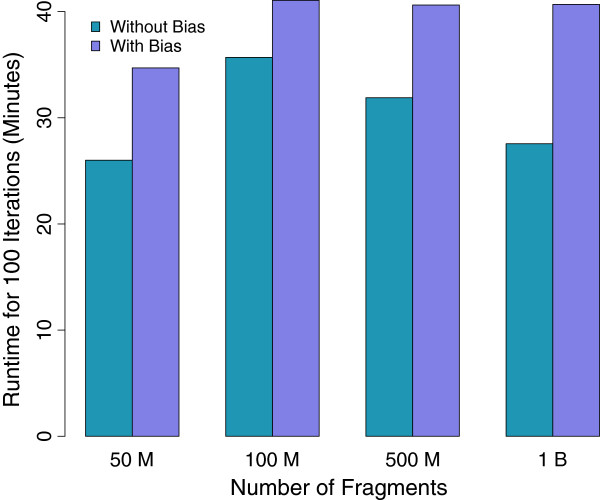
**Runtimes.** Average time required for 100 iterations on EC2 for different amounts of input data running on data simulated with (purple) and without (teal) sequence-specific bias. In the latter case, the timing is for iterations after the first 20, which require a constant 30 minutes to learn the bias model. The cluster size is scaled as 3 slave nodes (6 cores) for each 50 million fragments. The results show that eXpress-D running on Spark maintains constant runtime when resources are scaled linearly with the amount of the data.

In terms of speed and resource use, Figure 3 shows that eXpress-D can provide constant runtime if the number of nodes are increased linearly with the size of the input datasets. We found that with one CPU core per 1 million fragments, eXpress-D could execute 100 iterations in approximately 30 minutes without bias correction and 40 minutes with bias correction. There is also a constant 30 minute total overhead for learning the bias model during the first 20 iterations. Each run requires approximately 500 iterations to converge, meaning that only 4 hours would be required to process a billion fragments using 60 slave nodes (480 cores). This is only twice is long as is taken by the online EM algorithm of eXpress.

Although it is impossible to directly compare timings across different machines, we note that we previously found RSEM to be unable to complete the processing of more than 200 million fragments on a typical desktop server with 24 GB of RAM or 800 million fragments on a server with 512 GB of RAM [10], which is more than is available to many labs. Also, we show in Table 2 that eXpress, Cufflinks, and RSEM scaled with slopes that range from 1.8 minutes per million fragments (mpmf) to 27 mpmf on datasets that were successfully processed in [10]. Since eXpress-D runs in the cloud, it is not limited by the resources on a single machine and can easily scale to a billion reads with essentially no change in the time required.

**Table 2:** Slope of timing and resource curves

**Method**	**Runtime slope (mpmf)**	**Resource slope (cpmf)**
eXpress-D	0.05	0.12
eXpress	1.8	0
Cufflinks	6	0
RSEM	27	0

We computed the mean slope between the runtime samples from Figure 3 from of this manuscript and Figure 2(b) of [10] to compare the scaling of the four methods in units of minutes per million fragments (mpmf). While eXpress, Cufflinks, and RSEM were all run on the same machine fixed at 8 cores and 24 GB RAM, the resources of eXpress-D were increased at a rate of 0.12 cores per million fragments (cpmf), allowing it scale in approximately constant time.

## Conclusion

The distributed implementation of eXpress-D allows us to combine the full model of eXpress with the batch EM algorithm of RSEM to provide the best results in the least amount of time for large datasets. A simple extension to eXpress-D that also parallelizes the read alignment and pre-processing steps–similar to what is done in Myrna and Crowssbow[23]–would greatly improve performance and move the full analysis pipeline to the cloud.

As more genomic data moves to the cloud for storage, tools that are able to take advantage of distributed environments and frameworks–such as Spark–will become more widely used and help remove the barriers to large-scale integrative analysis of high-throughput sequencing projects.

### Availability and usage

eXpress-D and Spark are open source software that can be downloaded from their respective websites, http://github.com/adarob/express-d and http://spark.incubator.apache.org/. For ease of use, the eXpress-D source code includes a copy of a Spark script that allow users to launch, setup and manage EC2 clusters running Spark and HDFS. The script can be used to launch all nodes in the cluster using a customized Amazon Machine Image (AMI)–a type of templated operating system [24]–that is preloaded with eXpress-D source and binaries. Target and fragment datasets can then be loaded into HDFS or S3 for distributed execution. The eXpress-D wiki page includes more detail about using the script to launch clusters, as well as notes on cluster configuration and tuning.

## Competing interests

The authors declare that they have no competing interest.

## Authors’ contributions

AR developed the method. AR and HF implemented the method and analyzed the results. AR, HF, and LP wrote the manuscript. All authors read and approved the final manuscript.

## Supplementary Material

Additional file 1 **This script contains procedures for repeating our analyses in comparing **RSEM**, **Cufflinks**, and **eXpress**.** To reproduce our results, use in conjunction with the synthetic data available at bio.math.berkeley.edu/eXpress/simdata. Click here for file
